# Tendency to Ingest Foreign Bodies in Mentally Retarded Patients: A Case with Ileal Perforation Caused by the Ingestion of a Teaspoon

**DOI:** 10.1155/2016/8075432

**Published:** 2016-02-23

**Authors:** İhsan Yıldız, Yavuz Savaş Koca, Gökhan Avşar, İbrahim Barut

**Affiliations:** Department of General Surgery, Suleyman Demirel University School of Medicine, 32260 Isparta, Turkey

## Abstract

*Introduction*. Unintentional foreign body ingestion commonly occurs accidentally in children aged between 3 months and 6 years and at advanced ages or results from psychiatric disorders such as hallucination in patients with mental retardation. Most of the ingested foreign bodies are naturally discharged from the body but some of them may require surgical intervention.* Presentation of Case*. A 29-year-old mentally retarded female patient was admitted to the emergency service with a two-day history of abdominal pain, nausea, and vomiting. Physical examination revealed abdominal tenderness, defense, and rebound on palpation. Radiological examination revealed diffuse air-fluid levels and a radiopaque impression of a metal object in the right upper quadrant. The metal teaspoon causing ileal perforation was extracted by emergency laparotomy. On postoperative day 7, the patient was uneventfully discharged following a psychiatric consultation.* Discussion*. Foreign body ingestion can occur intentionally in children at developing ages and old-age patients, or adults and prisoners, whereas it may occur unintentionally in patients with mental retardation due to hallucination. However, repeated foreign body ingestion is very rare in individuals other than mentally retarded patients.* Conclusion*. Mentally retarded patients should be kept under close surveillance by surgeons and psychiatrists due to their tendency to ingest foreign bodies.

## 1. Introduction

Foreign body ingestion may occur intentionally or unintentionally. Unintentional foreign body ingestion commonly occurs accidentally in children aged between 3 months and 6 years and at advanced ages or results from psychiatric disorders such as hallucination in patients with mental retardation. Cases of intentional foreign body ingestion in adults and particularly in prisoners have also been reported in the literature [1–3].

Commonly ingested objects include coins, needles, pins, jewelry items, toy parts, teaspoons, nails, fish and chicken bones, items used for handling foods such as lollipop sticks, batteries, and dentures. These objects may be made of plastic, metal, or toxic materials [4, 5].

Most of the ingested foreign bodies are naturally discharged from the body within 4–6 days without causing any complications. Nevertheless, some of them may lead to serious complications in the gastrointestinal system that require surgical intervention, such as obstruction or perforation. These complications may also be associated with different clinical presentations such as nausea, vomiting, and acute abdomen depending on the localization and severity of the obstruction and perforation [5, 6].

Endoscopic approach may be the choice in the treatment of ingested foreign body removal via endoscopy, which is in the upper GI tract that is accessible, but in some cases, the objects in the lower gastrointestinal system are also inaccessible with endoscopy. Laparoscopic or surgical intervention is indicated in the presence of perforation or obstruction [2, 6]. Nevertheless, the adhesions caused by repeated laparotomies may lead to mechanical obstructions and perforation in the intestinal lumen [5–7].

In this report, we present a case that underwent repeated surgeries, initially due to duodenal perforation caused by the ingestion of a metal nail, secondly due to ileal perforation caused by the ingestion of a wood material, and finally due to ileal perforation caused by the ingestion of a metal teaspoon. This rare case was presented in line with the studies reported in the literature.

## 2. Presentation of Case

A 29-year-old mentally retarded female patient was admitted to the emergency service with a two-day history of abdominal pain, nausea, vomiting, and failure to eliminate feces or pass gas. Patient history revealed that the patient had undergone two surgeries due to repeated foreign body ingestion within the last 6 months. These two surgeries were performed due to duodenal perforation caused by the ingestion of a metal nail and ileal perforation caused by the ingestion of a wood material, respectively. Physical examination revealed that the temperature was 38.5°C, blood pressure was 90/60 mmhg, and heart rate was 96 beats/min, and the patient was present with muscular tenderness, defense, and rebound on palpation.

In radiological examination, direct abdominal graph at a standing position revealed diffuse air-fluid levels and a radiopaque impression suggestive of a metal object in the right upper quadrant.

**Figure 1:**
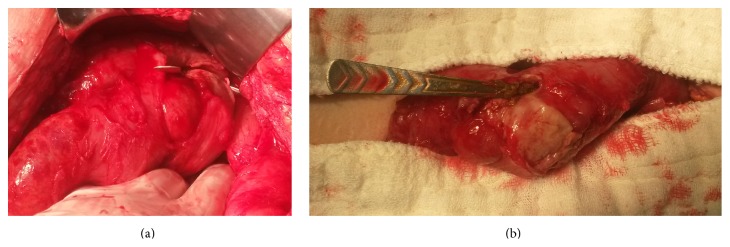
Teaspoon is shown.

In laboratory tests, white blood cell (WBC) count was 15,000/mcL (4000–8000/mcL) and other tests were in normal range (Figures 1(a) and 1(b)). A 15 cm segmental resection of the ileum, including the perforation site, with end-to-end anastomosis was performed and the teaspoon was removed. The abdomen was flushed with physiologic serum and the surgery was completed following the placement of a soft drain. On postoperative day 7, the patient was uneventfully discharged following a psychiatric consultation.

## 3. Discussion

Foreign body ingestion may occur intentionally in children at developing ages, old-age patients, or adults and prisoners, whereas it may occur unintentionally in patients with mental retardation due to hallucination [2, 3]. However, repeated foreign body ingestion is very rare in individuals other than mentally retarded patients [3, 7].

Our case was mentally retarded and had undergone surgery twice due to intestinal perforation caused by foreign body ingestion. Although a psychiatric consultation was performed prior to hospital discharge, no follow-up has been performed for the patient since hospital discharge.

Most of the ingested foreign bodies are naturally discharged from the body. The period of extraction mostly lasts for 5 days depending on the size and the structure of the ingested object. An orally ingested object passes through the oropharynx, pharynx, esophagus, cardia, pylorus, duodenum, small intestine, and colon, respectively, and then leads to obstruction and perforation at the anorectal level and consequently penetrates into the rectoanal region [4, 6]. The objects smaller than 6 cm in size and those with a structure thinner than 2 cm require no intervention and are naturally discharged from the body [5]. Moreover, in some cases, the ingested objects remain in the body without yielding any symptoms and unexpectedly lead to complications after a long period of time [4].

Although ingested foreign bodies are naturally discharged from the body, we consider that the foreign body in our patient was not naturally discharged since the capacity of bowel movements was limited due to the intraabdominal adhesions associated with previous surgeries and intestinal perforation was facilitated by these adhesions. In other words, while the previous perforations were caused by the ingestion of sharp items such as a nail, the last perforation occurred even as a result of a metal teaspoon.

About 10–20% of the ingested objects are extracted by endoscopy, whereas less than 1% of them require surgical intervention [3, 7]. Surgical intervention is indicated in the cases with perforation, obstruction, organ injury, and entrapment in surrounding tissues [2, 4–7]. Similarly, in our patient, surgical intervention was required due to the presence of perforation.

## 4. Conclusion

Mentally retarded patients constitute an important patient group for surgeons and psychiatrists due to their tendency to ingest foreign bodies and thus should be kept under close surveillance. Following the completion of their surgical treatment, these patients should also be evaluated for the mental problems that may lead to repeated foreign body ingestion.

## References

[1] Sai Prasad T. R., Low Y., Tan C. E., Jacobsen A. S. (2006). Swallowed foreign bodies in children: report of four unusual cases. *Annals of the Academy of Medicine Singapore*.

[2] Ricci S., Massoni F., Schiffino L., Pelosi M., Salesi M. (2014). Foreign bodies ingestion: what responsibility?. *Journal of Forensic and Legal Medicine*.

[3] Nijhawan S., Kumpawat S., Ashdhir P., Behl N., Jha A., Rai R. R. (2009). Impacted nail in duodenum: endoscopic removal with a novel magnetic foreign body retriever. *Endoscopy*.

[4] Cho E. A., Lee D. H., Hong H. J. (2014). An unusual case of duodenal perforation caused by a lollipop stick: a case report. *Clinical Endoscopy*.

[5] Başpinar I., Şahin S., Erdoğan G. (2010). Acute mechanical intestinal obstruction after ingestion of foreign bodies: a case report. *Ulusal Travma ve Acil Cerrahi Dergisi*.

[6] Mairose U. (2013). Intentional ingestion. *Deutsches Arzteblatt International*.

[7] Huang B. L., Rich H. G., Simundson S. E., Dhingana M. K., Harrington C., Moss S. F. (2010). Intentional swallowing of foreign bodies is a recurrent and costly problem that rarely causes endoscopy complications. *Clinical Gastroenterology and Hepatology*.

